# MAFB mediates the therapeutic effect of sleeve gastrectomy for obese diabetes mellitus by activation of *FXR* expression

**DOI:** 10.1590/1414-431X20187312

**Published:** 2018-05-28

**Authors:** Jian Xu, Yong Wang, Jiajun Yin, Min Yin, Mofei Wang, Jingang Liu

**Affiliations:** 1 Department of General Surgery, the Fourth Affiliated Hospital of China Medical University, Shenyang City, Liaoning Province, P.R. China; 2 Department of General Surgery, Affiliated Zhongshan Hospital of Dalian University, Dalian City, Liaoning Province, P.R. China

**Keywords:** Diabetes mellitus, Obesity, Sleeve gastrectomy, MAFB, FXR

## Abstract

Farnesoid X receptor (*FXR*) and related pathways are involved in the therapeutic effect of sleeve gastrectomy for overweight or obese patients with diabetes mellitus. This study aimed to investigate the mechanism of *FXR* expression regulation during the surgical treatment of obese diabetes mellitus by sleeve gastrectomy. Diabetic rats were established by combined streptozotocin and high-fat diet induction. Data collection included body weight, chemical indexes of glucose and lipid metabolism, liver function, and the expression levels of musculoaponeurotic fibrosarcoma oncogene family B (*MAFB*), *FXR*, and related genes induced by sleeve gastrectomy. Chang liver cells overexpressing MAFB gene were established to confirm the expression of related genes. The binding and activation of FXR gene by MAFB were tested by Chip and luciferase reporter gene assays. Vertical sleeve gastrectomy induced significant weight loss and decreased blood glucose and lipids in diabetic rat livers, as well as decreased lipid deposition and recovered lipid function. The expression of *MAFB, FXR*, and FXR-regulated genes in diabetic rat livers were also restored by sleeve gastrectomy. Overexpression of MAFB in Chang liver cells led to *FXR* gene expression activation and the alteration of multiple FXR-regulated genes. Chip assay showed that *MAFB* could directly bind with FXR promoter, and the activation of FXR expression was confirmed by luciferase reporter gene analysis. The therapeutic effect of sleeve gastrectomy for overweight or obese patients with diabetes mellitus was mediated by activation of FXR expression through the binding of *MAFB* transcription factor.

## Introduction

Diabetes mellitus (DM), also referred to as diabetes, is a common metabolic disorder characterized by aberrantly increased blood sugar level leading to severe complications such as cardiovascular disease, kidney disease, stroke, foot ulcers, and eye disease if left untreated (1). Type 2 DM, previously known as non-insulin-dependent DM, is the most common type of DM caused by insulin resistance, the condition in which human cells fail to properly respond to insulin (1). DM patients are frequently overweight or obese, and recent progress in an etiological study suggested that obesity and lack of physical exercise are the main causes of type 2 DM (2). Moreover, a current systematic review and meta-analysis confirmed that weight control by a healthy eating pattern, energy intake reduction, and regular physical activity should be encouraged as the primary prevention and treatment strategies for obese patients with type 2 DM (3). However, the specific mechanisms underlying the causative effect of obesity in diabetes development and the therapeutic efficacy of weight control for diabetics are not well known.

Sleeve gastrectomy (SG) has been applied as a promising surgical way of weight control for obese diabetics. A large-scale meta-analysis including 27 independent studies and 673 DM patients demonstrated that sleeve gastrectomy produced significant DM resolution and improvement of DM markers in most diabetics (4). Similar results were also observed by a mice model investigation showing that vertical sleeve gastrectomy (VSG) could lead to sustainable weight loss and relieve fatty liver and insulin resistance (5). In an animal model study using the University of California Davis-type 2 diabetes mellitus rat, VSG was reported to be effective not only for weight loss and DM resolution, but also for preventing the onset of type 2 DM (6). However, little is known about the molecular mechanisms mediating the roles of sleeve gastrectomy in causing weight loss and diabetes mellitus remission.

Farnesoid X receptor (*FXR*) acts as the sensor and nuclear receptor of bile acids, which activates FXR activity as nutrient signaling molecules (7). It has been well established that bile acids, the amphipathic detergent-like molecules as the end-products of cholesterol catabolism, could promote the solubilization of cholesterol and dietary lipids and are critically involved in lipid, cholesterol, and glucose metabolism (8,9). Bile acids binding with *FXR* induce the expression of fibroblast growth factor 15/19 to regulate bile acid synthesis, glycogen metabolism, and gallbladder filling (10). Consistent with the key roles of *FXR* in metabolism, *FXR* has been demonstrated to be associated with obesity-linked DM. For instance, *FXR* activity enhancement through agonist treatment or FXR gene overexpression leads to significantly decreased blood glucose levels in normal and diabetic mice, showing the critical function of *FXR* in glucose metabolism regulation (11). It is also worth mentioning that FXR agonists have been successfully applied as promising therapeutic agents for DM and other non-alcoholic fatty liver diseases (12). More importantly, the therapeutic value of VSG was revealed to be mediated by FXR signaling, thus leading to reduced body weight and improved glucose tolerance in DM mice (13). However, the mechanisms by which FXR was regulated during the substantial resolution of DM by sleeve gastrectomy deserve further investigation.

In this study, the effect of VSG on body weight, blood glucose, and lipid content, as well as on liver functions, was analyzed using a rat model of obese diabetes. To address the molecular mechanisms underlying the function of VSG in effectively inducing weight loss and diabetes symptom resolution, we predicted the musculoaponeurotic fibrosarcoma oncogene family B (MAFB) as one of the candidate transcription factors that might bind FXR promoter through bioinformatics analysis using JASPAR. A previous investigation showed that MAFB functions as a key regulator of islet α-cell activity and β cell maturation (14). Here, we investigated the influence of SG on MAFB expression, the regulation of FXR expression by MAFB, and also the downstream regulatory mechanisms, which provided novel insights into the mechanisms underlying the therapeutic effect of sleeve gastrectomy for obese patients with DM.

## Material and Methods

### Diet and animal models

**Figure 1.**
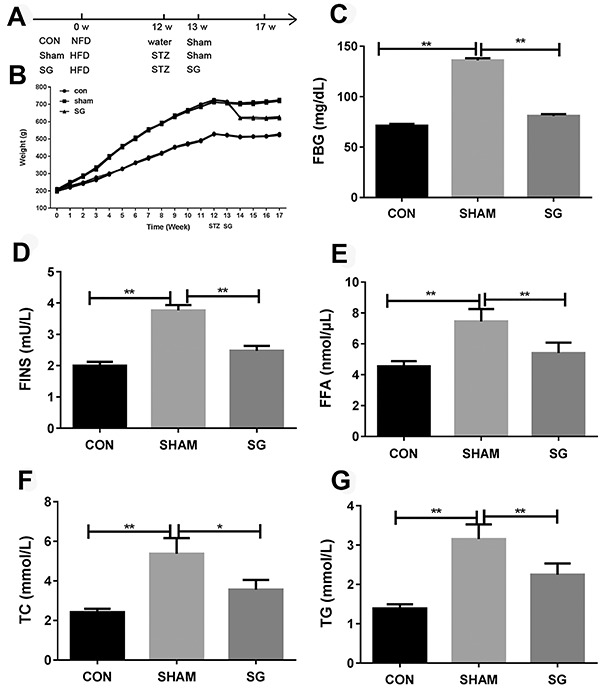
*A*, Experimental scheme and *B*, body weights of sleeve gastrectomy (SG), Sham, and control (CON) groups. Diabetes mellitus was established by combination of high-fat diet (HFD) and streptozotocin (STZ). SG and sham surgery were performed on diabetes mellitus rats. Rats fed with a normal-fat diet (NFD) and that underwent sham surgery were used as the control. *C*, Fasting blood glucose (FBG); *D*, fasting insulin (FINS); *E*, free fatty acid (FFA); F, total cholesterol (TC); *G*, triglyceride (TG). Data are reported as means±SD. *P<0.05, **P<0.01 (*t*-test).

Male Sprague-Dawley 8-week-old rats were housed individually in wire cages in the Animal Feeding Center of the Affiliated Zhongshan Hospital at the Dalian University and maintained on a 14-h light and 10-h dark cycle. The obese diabetic rats were established by the combination of high-fat diet (Guangdong Medical Laboratory Animal Center, China) and administration of streptozotocin (STZ) as previously described with minor modifications (6,15). Briefly, rats were first fed with a high-fat diet for 12 weeks, and then given a single intraperitoneal injection of 65 mg/kg of STZ. Three days after the STZ injection, the glucose content in the venous blood from the tail of STZ-treated rats was analyzed using a blood glucose meter (iChem-540, iCubio Company, China). Obese diabetic rats were defined by blood glucose level over 16.7 mmol/L and a weight of more than 395 g. Rats fed with normal-fat diet (Guangdong Medical Laboratory Animal Center) and given a single intraperitoneal injection of water were used as the control. One week later, the VSG or sham surgery was carried out separately on the obese diabetic rats. The experimental protocols of this study were approved by the Ethics Committee of the Affiliated Zhongshan Hospital of the Dalian University. Rats in this study were classified into three groups as shown in Figure 1A: the control group (Con) of normal rats fed with normal-fat diet in combination with injection of water and sham surgery, the Sham group (Sham) of diabetic rats that underwent sham surgery, and the sleeve gastrectomy (SG) group of diabetic rats that underwent VSG.

### Vertical sleeve gastrectomy and the sham surgery

VSG was performed on diabetic rats as previously described (6). Briefly, rats were fed with a liquid diet for consecutive 3 days before the VSG and sham surgery. Approximately 3–5% isoflurane was used to induce and maintain anesthesia of operated rats. A midline abdominal incision of approximately 3 cm was first made, followed by the transection of connective tissue attachments to rat liver and spleen, which allowed isolating the stomach. Approximately 70% of the stomach containing the entire fundus was removed and the remnant part in the tubular shape was left to connect the esophagus and pylorus. After being lavaged properly, the remnant of stomach was replaced into abdominal cavity. Finally, the abdominal cavity was closed. A combination of enrofloxacin (20 mg/kg) and meloxicam (2 mg/kg) was given to the operated rats for 14 consecutive days after surgery. The Sham group underwent a similar VSG as previously described and nothing was removed before suturing the abdominal incision of approximately 3 cm (6).

### Biochemical and physiological index measurements

Measurements of major biochemical and physiological indexes of the diabetic rats and the control group were performed as previously described (16). Briefly, after the three groups of rats were treated with the indicated diet and surgery, the levels of fasting blood glucose (FBG), free fatty acid (FFA), total cholesterol (TC), triglyceride (TG), aspartate aminotransferase (AST), and alanine aminotransferase (ALT) were determined by an auto-biochemical analysis apparatus supplied with the biochemical kit (Hitachi, Japan). The fasting insulin (FINS) level was measured using the insulin radioimmunoassay (RIA) kit (Beijing Atom High Tech, China) by an automated RIA-immunity analyzer (Xi'an Nuclear Instrument Factory, China).

### Lipid deposition analysis by oil red-O staining

Histological visualization of lipid deposition in liver sections of VSG or sham surgery-operated diabetic rats and control group was carried out using the oil red-O (Lipid Stain) kit (#ab150678; Abcam, UK) according to the manufacturer's instructions. Briefly, the rat liver sections were first incubated in propylene glycol for 2 min, in oil red-O solution for 6 min, and then in 85% propylene glycol for 1 min. After being rinsed 2 min with distilled water, the slides were incubated at 37°C with hematoxylin for 2 min, rinsed again with tap water and distilled water, and finally mounted using aqueous mounting medium under a coverslip. The lipid deposited in rat liver tissues was stained red and observed under microscopy.

### Quantitative RT-PCR and western blotting

The relative mRNA levels of related genes in the rat livers and Chang liver cells were analyzed by isolation of the total RNA and quantitative RT-PCR method (qRT-PCR). Briefly, total RNA samples from rat liver tissues or Chang liver cells were extracted with Trizol solution (Sigma-Aldrich, USA) following the manufacturer's instructions. The cDNA synthesis was carried out using approximately 2 µg RNA with EasyScript First-Strand cDNA Synthesis SuperMix kit (TransGen Biotech, China). The quantitative RT-PCR was performed by mixing 2 μL cDNA and 1 μL specific primers with the TransStart™ SYBR Green qPCR Supermix (TransGen Biotech) following the manufacturer's instructions. GAPDH was used as the internal control for the quantitation of gene expression. The sequences of primers used in this analysis are listed in Table 1. The relative protein abundance of MAFB and FXR in rat liver tissues and Chang liver cells was determined by western blotting using the anti-MAFB (#ab56242, Abcam, UK) and anti-FXR (#AB10304, Merck Millipore, Germany) antibodies according to the manufacturer's instructions.

**Table 1.** qPCR primers used in the study.

Gene	Primer
FXR forward	GGGCAACTGCGTGATGGA
FXR reverse	AGGAGGGTCTGCTGGTCTACA
MAFB forward	GCAGCAACGGTAGTGTGGA
MAFB reverse	TGACCTTGTAGGCGTCTCTCT
SHP-1 forward	GCCCTCTCTTCCTGCTTGG
SHP-1 reverse	GGTTGTGGTGGGTCTGGTG
SREBP-1 forward	CCAGCCTTTGAGGATAACCA
SREBP-1 reverse	CCGAAGCATCAGAGGGAGT
CYP7A1: forward	TGCCTTCTGTTACCGAGTGATGTT
CYP7A1 reverse	ACCGGCAGGTCATTCTCTACC
PEPCK forward	GCTGACAGACTCGCCCTATG
PEPCK reverse	CACCGTATCCGCTTCCG
G6Pase forward	CTCAGGAACGCCTTCTATGT
G6Pase reverse	GTGACGGGGAACTGTTTTATC
PPARa forward	AGAATCCACGAAGCCTACCT
PPARa reverse	AGAATCGGACCTCTGCCTC
FXR promoter forward	GGGGTACCAGGAGTCCCTCAGGCAGC
FXR promoter reverse	CCGCTCGAGTGTCATTTGTTTCCCGTCAC

### Cell culture and transfection

Human hepatocyte Chang liver cells were purchased from the Cell Bank of the Chinese Academy of Sciences (China) and cultured in DMEM medium containing 10% fetal bovine serum with 100 µg/mL streptomycin and 100 U/mL penicillin at 37°C in an incubator under 95% humidity and 5% CO_2_. The establishment of Chang liver cells overexpressing MAFB gene was finished according to the previously described protocol with minor modifications (17). Briefly, the human MAFB gene ORF was cloned into the pcDNA3.0 expression vector as described by the reference. For overexpression of MAFB gene, Chang liver cells were seeded in a culture dish and transfected with the MAFB-pcDNA3.0 construct using the Lipofectamine 2000 reagent (Invitrogen, USA) following the manufacturer's instructions. The pcDNA3.0 plasmid was used as the control plasmid DNA transfected with Lipofectamine 2000 reagent following the manufacturer's recommendations. The expression levels were finally checked by real-time PCR and western blotting 48 h after transfection.

### Chromatin immunoprecipitation (ChIP)

The binding of MAFB with FXR promoter was tested by chromatin immunoprecipitation (ChIP) using the ChIP Kit (#ab500, Abcam) following the manufacturer's instructions. The anti-MafA antibody ChIP Grade (#ab17976, Abcam) was used for immunoprecipitation. Finally, the binding was determined by PCR amplification of promoter sequence. The antibody against IgG was used as negative control.

### Transcriptional activity by luciferase reporter assays

The activation of FXR expression by MAFB was confirmed by luciferase-based reporter assay using the pGL3 promoter vector (#E1761, Promega, USA) following the manufacturer's instructions with reference to previously described protocol (18). Briefly, the wild-type FXR promoter as well as the mutant version of FXR promoter with mutation of the predicted binding sites were separately cloned into the pGL3 promoter vector. The FXR promoter was amplified using primers named FXR promoter forward and reverse as in Table 1. HEK293 cells purchased from the Cell Bank of the Chinese Academy of Sciences were cultured in DMEM containing 10% fetal calf serum at 37°C under 95% humidity and 5% CO_2_. The transfection of HEK293 cells with the pGL3 promoter vectors containing the wild-type or mutant FXR promoter was carried out using the Fugene HD transfection reagent (Roche, USA) following the manufacturer's instructions. The cells were then lysed using passive lysis buffer (Promega) 48 h after transfection. The luciferase enzyme activity was presented as fold-change relative to the vehicle control.

### Statistical analysis

Statistical analysis was performed using the SPSS software package (version 18.0, SPSS). The significance of differences was statistically tested by the Student's *t*-test using data from at least three biological replicates. Significant differences were defined by a P value <0.05.

## Results

### VSG induced weight loss and decreased blood glucose and lipids in diabetic rats

The body weights of rats were measured each week from the beginning of this study, showing that the high-fat diet induced a significant weight increase in the Sham and SG groups before surgery, compared to the control group. The sleeve gastrectomy significantly decreased body weights of diabetic rats in the SG group (Figure 1A). Consistent with the change of weight, blood glucose, and lipid analysis also demonstrated that FBG, FINS, FFA, TC, and TG of the SG group were greatly lowered compared to the Sham group (Figure 1B-G), showing the effective function of VSG in causing weight loss and diabetes mellitus resolution via improved glucose and lipid metabolism.

### VSG improved liver function of diabetic rats

**Figure 2.**
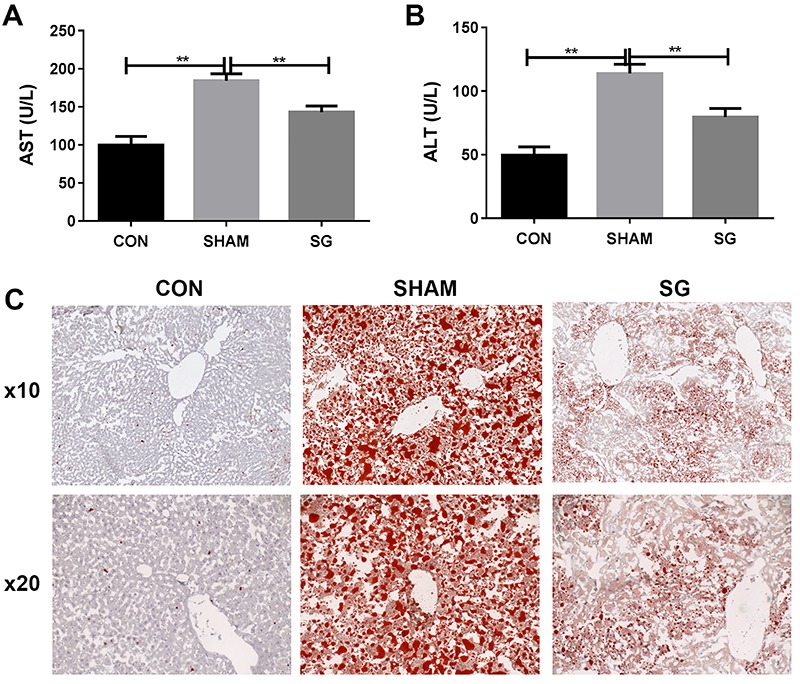
Sleeve gastrectomy improved liver function of diabetic rats. *A* and *B*: Levels of aspartate aminotransferase (AST) and alanine aminotransferase (ALT) in sleeve gastrectomy (SG), sham, and control (CON) rats. *C*: Lipid deposition in liver tissues of SG-operated diabetic rats. Tissues were stained red by the oil red-O method. Data are reported as means±SD. **P<0.01 (*t*-test).

The levels of two common liver function markers, AST and ALT, were remarkably elevated in the sham group, compared with the control group, but VSG effectively recovered the AST and ALT levels (Figure 2A and B). Results of the oil red-O dyed-tissues showed that the fat deposition in the STZ-induced diabetic rats was greatly enhanced compared with the control group, but sleeve gastrectomy significantly repressed lipid deposition in diabetic rat liver tissues (Figure 2C). These results indicated that sleeve gastrectomy could effectively recover the liver functions of diabetic rats, showing the important mediating role of the liver organ during DM therapy by surgery.

### Recovered MAFB and FXR expression by VSG

**Figure 3.**
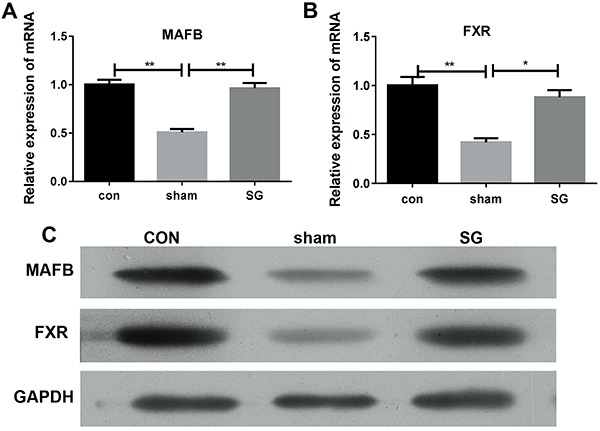
*A*, MAFB and *B*, FXR mRNA levels in sleeve gastrectomy (SG), sham, and control (CON) rats measured by quantitative RT-PCR. *C*: MAFB and FXR protein levels determined by western blotting. GAPDH was applied as the internal control. MAFB: musculoaponeurotic fibrosarcoma oncogene family A; FXR: farnesoid X receptor; GAPDH: glyceraldehyde-3-phosphate dehydrogenase. Data are reported as means±SD. *P<0.05 and **P<0.01 (*t*-test).

To explore the molecular mechanisms underlying the physiological effects of VSG, the MAFB protein, which regulates islet α-cell activity and β cell maturation (14), was predicted as a possible regulator of FXR expression (data not shown). Our results showed that the mRNA levels of both MAFB and FXR were greatly decreased in the liver tissues of sham-operated diabetic rats, but VSG recovered the MAFB and FXR mRNA levels to the degrees comparable to those of the control group (Figure 3A and B). Consistently, the decreased protein abundance of MAFB and FXR in diabetic rat livers was also restored by VSG (Figure 3C). The changes of MAFB and FXR expression in diabetic rats, which are strictly correlated with glucose metabolism and liver function, indicated that these two proteins might be key players mediating DM resolution by sleeve gastrectomy.

### Modulation of FXR-regulated signaling components by sleeve gastrectomy

**Figure 4.**
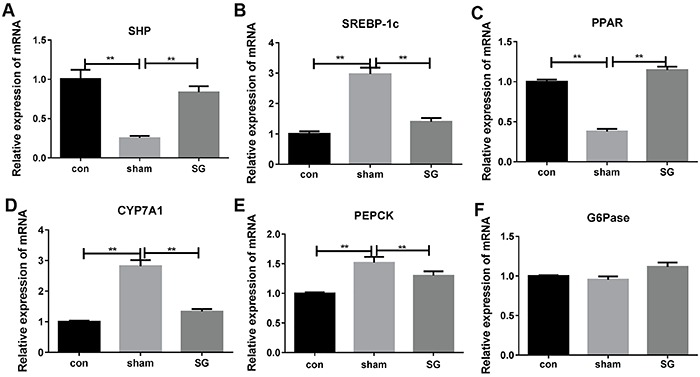
mRNA levels of six FXR-regulated genes, SHP (*A*), SREBP (*B*), PPAR (*C*), CYP7A1 (*D*), PEPCK (*E*), and G6pase (*F*) in livers of diabetic rats that underwent sleeve gastrectomy (SG) or sham surgery analyzed by quantitative RT-PCR. GAPDH was applied as the internal control in qRT-PCR analyses. Con: control group; SHP-1: small heterodimer partner 1; SREBP-1: sterol regulatory element binding protein-1; PPARα: peroxisome-proliferator-activated receptor α; CYP7A1: cholesterol 7α-hydroxylase gene (CYP7A1); PEPCK: phosphoenolpyruvate carboxykinase; G6Pase: glucose-6-phosphatase. Data are reported as means±SD. **P<0.01 (*t*-test).

To further investigate the role of FXR in VSG-regulated glucose metabolism and diabetes mellitus progression, the expression levels of six key signaling components regulated by FXR in VSG-operated diabetic rat livers were also measured by quantitative RT-PCR method. The mRNA level of small heterodimer partner 1 (SHP-1) was significantly decreased in diabetic rats but recovered by VSG (Figure 4A). On the contrary, the expression of sterol regulatory element binding protein-1 (SREBP-1) was remarkably increased in diabetic rat livers compared with the control group, but markedly repressed by the VSG (Figure 4B). The expression level of peroxisome-proliferator-activated receptor α (PPARα) showed the same alteration in these groups as SHP-1 (Figure 4C). Also, the expression of cholesterol 7α-hydroxylase gene (CYP7A1) exhibited correlative alteration in the sham and SG-operated diabetic liver rats compared with the control group (Figure 4D). Moreover, our results showed that the phosphoenolpyruvate carboxykinase (PEPCK) expression was contrarily influenced by the sham surgery and VSG in diabetic rats (Figure 4E). No significant change of the glucose-6-phosphatase (G6Pase) was detected in this assay (Figure 4F). The significant and correlated expression changes of these FXR-regulated genes provided multiple lines of evidence suggesting the strong regulatory functions of FXR in resolution of DM by VSG.

### Overexpression of MAFB regulated FXR-associated signaling cascades

**Figure 5.**
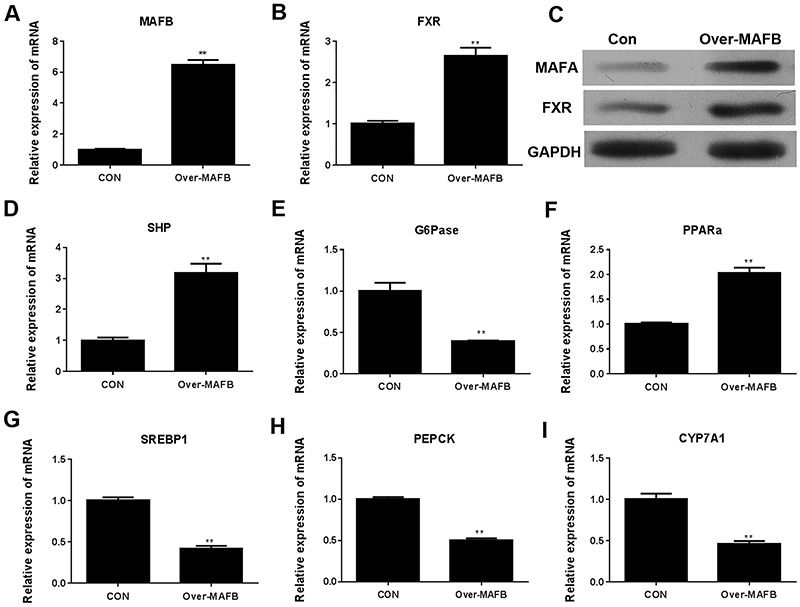
mRNA levels of MAFB (*A*) and FXR (*B*) in Chang liver cells overexpressing MAFB gene (over-MAFB) were analyzed by quantitative RT-PCR and by western blotting (*C*). The mRNA levels of SHP (*D*), G6Pase (*E*), PPARα (*F*), SREBP (*G*), PEPCK (*H*), and CYP7A1 (*I*) were analyzed by quantitative RT-PCR. GAPDH was applied as the internal control in qRT-PCR analysis. Con: control group; FXR: farnesoid X receptor; SHP: small heterodimer partner; SREBP-1: sterol regulatory element binding protein-1; CYP7A1: cholesterol 7α-hydroxylase gene (CYP7A1); PEPCK: phosphoenolpyruvate carboxykinase; G6Pase: glucose-6-phosphatase; PPARα: peroxisome-proliferator-activated receptor α. Data are reported as means±SD. **P<0.01 (*t*-test).

To investigate the role of MAFB in regulating FXR expression and downstream signaling cascades, Chang liver cells were transiently transfected with pcDNA3.0 plasmid containing the open-reading frame (ORF) of MAFB, and the original pcDNA3.0 vector was used as the control. The overexpression of MAFB in Chang liver cells were confirmed by both the quantitative RT-PCR and western blotting (Figure 5A and C). More importantly, the expression of FXR was also greatly enhanced by overexpression of MAFB in Chang liver cells, showing the direct activating effect of MAFB on FXR expression (Figure 5B and C). To further explore the role of MAFB in inducing FXR expression, these four FXR-regulated genes shown in Figure 4D to I, together with G6Pase and PPARα, were also analyzed. Our results demonstrated that the expression of SHP, G6Pase, PPARα, SREBP, PEPCK, and CYP7A1 were all greatly altered by the overexpression of MAFB in Chang liver cells. These cells clearly showed the potent ability of MAFB in regulating FXR and related molecular processes in human liver cells.

### MAFB binded with FXR promoter to induce FXR expression

**Figure 6.**
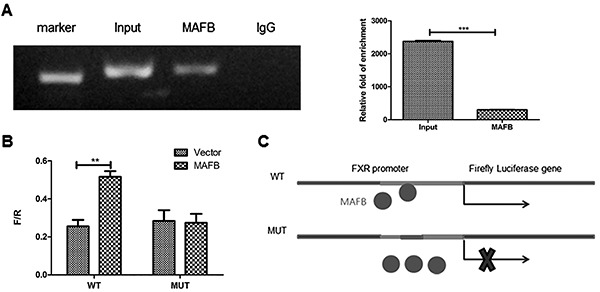
*A*, The binding of MAFB with FXR promoter was analyzed by Chip assay using antibodies against MAFB protein (left), and statistical analysis was based on data from three biological replicates (right). IgG was used as the negative control. *B,* Activation of FXR gene expression by MAFB binding by luciferase reporter assay. *C,* Schematic demonstration of the luciferase reporter assay. MAFB: musculoaponeurotic fibrosarcoma oncogene family; F/R: Firefly luciferase/Ranilla luciferase; WT: wild type; MUT: mutant; FXR: farnesoid X receptor. Data are reported as means±SD. **P<0.01, ***P<0.001 (*t*-test).

The RT-PCR amplification following Chip assay demonstrated that MAFB protein directly associated with FXR promoter in HEK293 cells (Figure 6A). For the investigation of activating ability of MAFB binding for FXR expression, luciferase reporter assay was also performed in HEK293 cells, and results showed that the luciferase gene was significantly activated by the binding of MAFB with FXR promoter, while the mutant version showed no significant activation of reporter gene expression. These results clearly demonstrated that MAFB was a key transcription factor responsible for the activation of FXR expression by directly binding the FXR gene promoter.

## Discussion

DM is a severe metabolic disorder characterized with dysregulated glucose metabolism and previous investigations showed strong correlation of DM with obesity (2). Although weight control by surgical operations like VSG has been shown to be an effective method for treatment of DM patients with severe obesity (4), the molecular mechanisms underlying this method are still poorly understood. As introduced above, FXR was shown to be an important mediator of DM development by regulating glucose and lipid metabolism and was revealed to be involved in the therapeutic effect of VSG (12,13). However, the regulation of *FXR* gene expression during DM progression is not well investigated, especially the transcription factors responsible for the activation of FXR expression associated with DM. Using bioinformatics analysis, *MAFB* gene was found to be a potential transcription factor regulating *FXR* gene expression. In this study, we reported that the expression of MAFB was strictly correlated with FXR and multiple FXR-regulated genes in diabetic rats that underwent VSG.

The enhancement of FXR expression by MAFB was further confirmed by the overexpression of MAFB gene in Chang liver cells. This regulation was supported by the expression of downstream genes controlled by FXR, including SHP-1 as the nuclear receptor induced by FXR and involved in bile acid biosynthesis (19), SREBP-1, which is associated with lipid metabolism and negatively regulated by FXR (20), PPARα, which controls SREBP activity and lipid synthesis (21), CYP7A1, which acts as another important regulator of bile acid metabolism inhibited by both FXR and SHP (19,22), and also PEPCK, which regulates the rate-limiting step of hepatic gluconeogenesis and is activated by FXR (23). G6Pase acts as regulator of hepatic gluconeogenesis (24), and we noticed that its expression in rat livers exhibited no significant alteration as shown in Figure 4F, which might be due to the functional abundance of multiple metabolism regulators (25) or the specific expression and functioning of G6Pase isozymes (26). Moreover, by Chip assay and luciferase-based reporter gene analysis, we found that the activation of FXR expression was mediated by the direct binding of MAFB with FXR promoter. These results revealed a novel mechanism of FXR-mediated DM development and treatment by sleeve gastrectomy, through the transcription factor activity of MAFB. Combined with roles of FXR in bile acid, glycogen, and lipid metabolism (10,27), these findings indicated that surgery resulted in FXR expression change, downstream signaling activation, and metabolic adaptions. This discovery provided important insight into the molecular pathogenesis of DM, as well as the therapeutic effect of sleeve gastrectomy.

MAFB protein, as a basic leucine-zipper-containing transcription factor, was first identified as the interacting partner of the DNA-bound Ets-1 protein and involved in erythroid differentiation (28). Further investigation showed that MAFB plays roles in multiple biological and pathological process such as monocytic differentiation, osteoclast differentiation, self-renewal of differentiated functional macrophages, myeloid commitment divisions of hematopoietic stem cells, respiratory rhythmogenesis, and fatal central apnea (29). More importantly, MAFB was later found to be a potent regulator of pancreatic α-cell activity and β cell maturation (14,30). For instance, MAFB was shown to regulate cell type-specific glucagon gene expression and associated with islet cell development and obesity (31). One recent study using a mice model reported that MafB deficiency in hematopoietic cells could lead to a greatly increased percentage of body fat, thus accelerating the development of obesity (31). Considering the close association of islet beta cell failure in obesity-associated diabetes (32), it is reasonable to predict the involvement of MAFB in DM development because of the beta cell compensation for insulin resistance. Not surprisingly, MAFB was found to possess a protective role for diabetic nephropathy by regulation of multiple pathways including antioxidative enzymes and Notch pathways (33). In this study, we discovered the direct link between MAFB-regulated target gene expression and DM development and treatment, which could lay down key research basis for the understanding of diabetes pathogenesis and novel therapy development. It should be noted that MAFB, as a transcription factor, might regulate the expression of multiple genes besides FXR, suggesting that further study of its downstream regulatory pathways could broaden our understanding of DM development. In consideration of the several functions of MAFB and FXR in metabolism, the possible involvement of MAFB-regulated FXR expression in other metabolic disease deserves further investigation.

Moreover, the application of diabetic rat or mouse models induced by high-fat diet and STZ, an antibiotic causing the destruction of pancreatic islet β-cells, has been accepted as a feasible way to test clinical therapeutic effects and underlying mechanism of VSG for diabetic patients (34). Both the mice and rats are sensitive to the pancreatic β-cell cytotoxic effects induced by STZ treatment (34), but rats are more suitable for surgical operation due to body shape and thus were applied in this study. By strictly following the widely proven protocol, we successfully established the obese DM rat model, which was confirmed by a change of the body weight and a number of physiological indexes associated with glucose and lipid metabolism and liver function after VSG. This establishment provided the basis for the mechanism investigation of DM combined with obesity and confirmed the applicability of this model for obese DM studies.

In summary, we reported the characterization of MAFB as a novel transcription factor responsible for the activation of FXR expression and downstream pathway during DM resolution induced by sleeve gastrectomy. The transcription factor activity of MAFB for FXR expression was confirmed by expression analysis in diabetic rat livers and Chang liver cells, combined with the binding and luciferase reporter gene assays. The discovery provides meaningful insight into the molecular processes underlying DM development, as well as the therapeutic effects of sleeve gastrectomy.
